# Return to performance following severe ankle, knee, and hip injuries in National Basketball Association players

**DOI:** 10.1093/pnasnexus/pgac176

**Published:** 2022-09-04

**Authors:** Garrett S Bullock, Tyler Ferguson, Amelia H Arundale, Chelsea Leonard Martin, Gary S Collins, Stefan Kluzek

**Affiliations:** Department of Orthopaedic Surgery, Wake Forest School of Medicine, Winston–Salem, NC 27157, USA; Centre for Sport, Exercise and Osteoarthritis Research Versus Arthritis,University of Oxford, Nottingham NG7 2UH, UK; Big Data Institute, University of Oxford, Oxford OX3 7LF, UK; Red Bull Athlete Performance Center, 5303 Thalgau, Austria; Icahn School of Medicine, Mount Sinai Health System, New York, NY 10029, USA; ATI Physical Therapy, Greenville, SC 29605, USA; Centre for Statistics in Medicine, University of Oxford, Oxford OX2 6UD, UK; Oxford University Hospitals NHS Foundation Trust, Oxford OX3 9DU, UK; Centre for Sport, Exercise and Osteoarthritis Research Versus Arthritis,University of Oxford, Nottingham NG7 2UH, UK; Nuffield Department of Orthopaedics, Rheumatology, and Musculoskeletal Sciences, University of Oxford, Oxford OX3 7LD, UK; University of Nottingham, Nottingham NG7 2RD, UK

**Keywords:** data scraping, performance analytics, minutes, points

## Abstract

The purpose of this study was to compare basketball performance markers 1 y prior to initial severe lower extremity injury, including ankle, knee, and hip injuries, to 1 and 2 y following injury during the regular National Basketball Association (NBA) season. Publicly available data were extracted through a reproducible extraction computed programmed process. Eligible participants were NBA players with at least three seasons played between 2008 and 2019, with a time-loss injury reported during the study period. Basketball performance was evaluated for season minutes, points, and rebounds. Prevalence of return to performance and linear regressions were calculated. A total of 285 athletes sustained a severe lower extremity injury. A total of 196 (69%) played for 1 y and 130 (45%) played for 2 y following the injury. A total of 58 (30%) players participated in a similar number of games and 57 (29%) scored similar points 1 y following injury. A total of 48 (37%) participated in a similar number of games and 55 (42%) scored a similar number of points 2 y following injury. Fewer than half of basketball players who suffered a severe lower extremity injury were participating at the NBA level 2 y following injury, with similar findings for groin/hip/thigh, knee, and ankle injuries. Fewer than half of players were performing at previous preinjury levels 2 y following injury. Suffering a severe lower extremity injury may be a prognostic factor that can assist sports medicine professionals to educate and set performance expectations for NBA players.

Significance Statement Fewer than half of basketball players who suffered a groin/hip/thigh, knee, or ankle injury played in the National Basketball Association 2 y following injury. Fewer than half of these players returned to similar performance following a severe injury. The data and code are freely accessible, allowing sport professionals to directly compare individual players to similar National Basketball Association players returning to sport following a severe lower extremity injury. These data and direct comparisons can be used to educate and set performance expectations for players, teams, and organization as they return to sport following a severe injury.

**Abbreviations**
ACL: Anterior Cruciate Ligament
AGE: Athlete Game Exposure
NBA: National Basketball Association

## Introduction

Injuries in professional male basketball are a significant problem (1–3). Direct costs of injuries impact the National Basketball Association (NBA) an estimated $350 million in lost revenue each season (4). Each injury affects an individual player, but also has consequences for the whole organization, with indirect costs extending to utilization of the medical team, wages, and revenue (5). Further, injuries are known to affect performance at an individual level (6–8), but a greater injury burden is associated with worse team performance (5). As a result, many clinicians in high performance sport are frequently asked to predict not only how long until an athlete returns to play, but how long until they return to their preinjury level of performance (9). Due to the monetary implications and multilayered effect of injuries, there is a great deal of pressure to estimate the performance implications of specific basketball injuries (1, 10).

Studies have examined the effect of specific injuries, such as anterior cruciate ligament (ACL) injuries (6, 7) or Achilles ruptures (8), on performance in professional basketball. For example, NBA players who sustained an Achilles rupture demonstrated a decrease of 4.38 player efficiency rating (PER) 2 y following injury (8). Although ACL and Achilles injuries present a significant burden in sport, ACL injuries are not the most common knee injuries in basketball and Achilles ruptures are rare (1, 10). Clinicians, coaching staff, and team managers would benefit from knowledge regarding the most common severe injuries and their impact on performance.

The contribution of players to overall team performance varies in terms of minutes played, points per season, and season rebounds (6–8). Therefore, a severe injury may have implications across multiple performance parameters. Currently, it is unclear how common severe basketball injuries associate with NBA performance the season following a severe injury. Understanding if suffering a severe lower extremity injury is a prognostic factor in relation to performance would assist clinicians to communicate realistic return to performance expectations to athletes, coaches, and organizations. Therefore, the purpose of this study was to compare basketball performance markers 1 y prior to initial severe lower extremity injury, including ankle, knee, and hip injuries, to 1 and 2 y following injury during the regular NBA season.

## Materials and methods

### Study design

This is a retrospective self-controlled prepost study incorporating publicly available data. The Strengthening the Reporting of Observational Studies in Epidemiology for Sport Injury and Illness Surveillance (STROBE-SIIS) were followed (11). Data that are available through the internet (i.e. public) were extracted through a procedure called “data scraping” (12). These data have previously been published (3, 13). Three online repositories were employed to extract these data:

1. “Pro Sport Transactions,” a database that processes all professional sport activities in North America. These activities include draft picks, trades, free agent signings, and injuries (https://www.prosportstransactions.com). Within the website, the basketball tab, under basketball transactions was data scraped, beginning with https://www.prosportstransactions.com/basketball/Search/SearchResults.php?Player = &Team = &BeginDate = &EndDate = &PlayerMovementChkBx = yes&Submit = Search.
2. Basketball-Reference.com, a website that updates and continuously deposits all NBA performance data (https://www.basketball-reference.com). Each NBA player season performance statistics were extracted for every included season. https://www.basketball-reference.com/leagues/NBA_2022_totals.html.
3. The official NBA website, which reports NBA performance and injury data (https://stats.nba.com). The NBA performance metrics were extracted from the exact website https://www.nba.com/stats/players/. The performance metrics were compared to “basketball-reference.” Return to game dates were compared to “Pro Sport Transactions.”

### Data extraction

The website https://www.prosportstransactions.com was used as the initial data extraction website. Data extract was performed on 2019 December 1. The timeline parameters of data extraction were from the beginning of website collection through 2019 December 1. Data from the 2020 season (i.e. the COVID-19 season) were not included within these analyses (3, 13). Basketball-Reference.com and stats.nba.com websites were used to data scrape additional data. Data included games played, games missed, playing position, and season performance statistics, including minutes played, points scores, and rebounds. A custom R package was created (“NBAinjuries”) to extract and cross-reference the NBA game and performance data. For code and data, refer to the GitHub R Package “tylerferguson/NBAinjuries.”

### Data cleaning

Deviations in data were observed through quality checks, including missed days and team names, and incomplete dates from data inception through the 2007 season. These data were inconsistent due to technological issues at the beginning of the websites inceptions. Data before 2008 were excluded (3, 13). The website https://www.prosportstransactions.com demonstrated reliable injury date recording but inconsistent injury date of recovery. The https://www.prosportstransactions.com were harmonized with https://stats.nba.com and https://www.basketball-reference.com data at the individual game level to determine time to injury recovery. As practice dates were not included in these data, the first game played following injury was adopted for date of injury recovery (i.e. resolution). Automated internal validation checks were performed to assess for values that are no possible such as missing or negative days to injury recovery were not included (3, 13).

### External validation

Data were externally validated through two independent examiners. A total of 180 data points were randomly externally validated through a random number generator (Appendix S2). Injury and body part data were compared to other public internet based data including: NBA.net, ESPN.com, and individual NBA organization websites (3, 13). Measure of agreement was 44.7% for exact date, 84.1% for missed games, and 95.6% for injury site (Appendix S2). The injury date low measure of agreement was determined to be due to that the scraped data recounted the first game missed from injury. On the other hand, the external data reported the exact date. The differences between the scraped and external data imply the external data would report the exact training or practice date, while the scraped data reported data only at the game level. Further discrepancies between data may be due to an NBA player being reactivated on the playing roster, but not receiving playing minutes. While NBA players may have been reactivated on the playing roster, the player may not be ready to perform in game (3, 13).

### Participants

NBA players were appropriate if they were 18 y and older, and played at least three seasons between 2008 and 2019, with a severe time-loss lower extremity (ankle, knee, or hip) injury reported during the study period. Exclusion criteria were as follows: (1) less than three seasons of experience due to their difference in injury risk (13); (2) injured and did not play in the season prior to injury; and (3) injured and did not play in the season following return to sport.

The index season was identified as the season where the severe lower extremity injury occurred. If a severe lower extremity injury resulted in missing games in the next season, this was still considered part of the index season. The season prior to the index season was defined as the season preceding the severe lower extremity injury. The athlete could not sustain more than a minor injury during this season. The season following the index injury (Year 1) was determined after the athlete had returned to sport, and did not suffer a recurrent or subsequent injury within 14 d following return to sport (14).

#### Exposure definition

Athlete exposure was calculated through athlete game exposures (15). Player minutes was included as a performance marker, due to potential changes in minute play following a severe injury, or changes in overall player performance.

#### Injury and illness definitions

An injury was demarcated as tissue damage that transpired during any NBA session and resulted in at least a timeloss of one game (2, 11, 16). Data were only included from the first regular season game to the last regular season. Other player activities, including preseason, playoffs, and off-season injuries were not included. These data were not included due to the data irregularity and some injuries could not be validated as being sustained during basketball exposure. Injuries were also stratified by body part (17). Injury severity was stratified into Slight (1 game), Minor (2 to 3 games), Moderate (4 to 13 games), or Severe (14+ games) (11, 18).

### Outcomes

Specific performance markers during regular NBA season included: games started, season minutes, season points, points per game, season field goals attempted, and season rebounds.

### Statistical analyses

Prior to data analysis, missing data were assessed. Missing data were 0% for index season, the season prior to the index, and the season following the index season. However, 33% of basketball players demonstrated missing performance data (minutes played, points in a season, and field goals attempted) 2 y following index season. Reasons for the missing performance data were explained through being released from an NBA team and thus were determined to be missing not at random. As multiple imputation is not suggested for missing not at random data, complete case analyses were performed.

Continuous variables were summarized as means (SD) and count data were summarized as percentages. ANOVA was performed to assess return to sport time between groin/hip/thigh, knee, and ankle injuries. Performance measurements were stratified by ankle, knee, and hip. Prevalence of athletes that returned to preinjury performance with 95% CIs were calculated (19).

Linear regression models were fit to understand the prognostic association of season basketball performance (minutes played, points scored, and rebounds) following severe lower extremity injury. The first season and second season performance following return to sport after a primary severe injury were each included individually in the regression model as the explanatory variables, with the season prior to the primary severe injury as the comparison. Results were presented as regression coefficients with 95% CI. Sensitivity analyses were performed including severe knee injuries only and nonlinear analyses with restricted cubic splines. Points scored and rebounds also controlled for minutes played as a secondary analysis. All analyses were performed in R version 4.02 (R Core Team, 2013).

## Results

### Descriptive and performance statistics

A total of 285 athletes sustained a severe lower extremity injury, with 196 (69%) athletes playing at least 1 y following the injury (Table 1, flow diagram in Appendix S1). There was no statistical difference in number days to return to sport between groin/hip/thigh [227 (88)], knee [260 (160)], or ankle [260 (77)] basketball players (*P* = 0.289). There was a descriptive decrease in basketball performance markers in seasons following a primary severe lower extremity injury compared to the season before a primary lower extremity injury (Table 2; Appendix S3).

**Table 1.** NBA players demographics.

**Variable**	**Severe lower extremity injury** **(*n* = 196)**	**Severe groin/hip/thigh injury (*n* = 39)**	**Severe knee injury (*n* = 111)**	**Severe ankle injury (*n* = 46)**
Age at injury (years)	26.9 (3.9)	26.3 (3.1)	27.1 (4.0)	27.1 (4.2)
Seasons played in the NBA	8.0 (2.7)	7.8 (2.6)	7.9 (2.7)	8.5 (2.7)
BMI (kg/m^2^)	25.1 (1.7)	25.1 (1.9)	25.0 (1.7)	25.0 (1.8)
Position				
Guard	39%	45%	34%	46%
Forward	41%	38%	45%	35%
Center	20%	17%	21%	19%

All descriptive statistics are reported as mean (SD) or percentage.

**Table 2.** NBA players performance statistics for 1 y prior and 1 and 2 y following severe injury.

**Variable**	**Severe lower extremity injury (*n* = 196)**		
	**1 y prior**	**1 y following**	**2 y following**
Games played	65 (17)	51 (25)	56 (22)
Games started	40 (29)	26 (29)	33 (30)
Season minutes played	1730 (750)	1243 (883)	1430 (871)
Minutes played per game	25.8 (8.1)	21.6 (9.2)	23.3 (9.2)
Season points	761 (468)	563 (506)	687 (552)
Points per game	11.2 (5.8)	9.3 (6.2)	10.8 (6.8)
Season rebounds	297 (200)	223 (208)	260 (216)
Rebounds per game	3.6 (2.4)	2.7 (2.5)	4.2 (2.9)

Results are reported as mean (SD).

A total of 33% of basketball players who suffered a severe lower extremity injury did not play a second season following injury. By injury, 33% of basketball players who suffered a severe ankle injury, 33% that suffered a severe knee injury, and 30% that suffered a severe groin/hip/thigh injury, did not play a second season.

Only 30% (95% CI: 23, 36) of basketball players who suffered a lower extremity injury, returned to their preinjury number of games 1 y following injury and only 37% (95% CI: 29, 45) of players return to their preinjury number of games in 2 y (Table 3). A similar trend was seen for all metrics. For most markers, less than 30% of players return to their preinjury level of performance within 1 y of severe injury. More athletes returned to their preinjury level of performance within 2 y. However, in most cases, this was still under 50% of players (Table 3).

**Table 3.** Prevalence of NBA players reaching preinjury performance stratified by year.

	**Games played**	**Games started**	**Season minutes played**	**Minutes played per game**	**Season points**	**Points per game**	**Season rebounds**	**Rebounds per game**
1 y following severe injury								
Severe lower extremity injury (*n* = 196)	30% (23, 36)	32% (27, 38)	28% (21, 34)	26% (20, 32)	29% (23, 35)	29% (23, 35)	29% (23, 35)	29% (23, 35)
Severe groin/hip/thigh injury (*n* = 39)	33% (19, 48)	33% (19, 48)	33% (19, 48)	28% (14, 42)	33% (19, 48)	33% (19, 48)	33% (19, 48)	33% (19, 48)
Severe knee injury (*n* = 111)	32% (24, 41)	32% (24, 41)	25% (17, 33)	21% (13, 28)	27% (19, 35)	25% (17, 33)	27% (19, 35)	26% (18, 34)
Severe ankle injury (*n* = 46)	20% (8, 31)	28% (15, 41)	28% (15, 41)	37% (23, 51)	30% (17, 44)	35% (21, 49)	28% (15, 41)	33% (19, 46)
2 y following severe injury								
Severe lower extremity injury (*n* = 130)	37% (29, 45)	45% (37, 54)	34% (26, 42)	31% (23, 39)	36% (27, 44)	42% (33, 50)	36% (28, 44)	35% (27, 43)
Severe groin/hip/thigh injury (*n* = 27)	26% (9, 42)	41% (22, 59)	27% (10, 43)	26% (9, 42)	33% (16, 51)	41% (22, 59)	33% (16, 51)	33% (16, 51)
Severe knee injury (*n* = 73)	41% (30, 52)	44% (33, 55)	32% (21, 42)	27% (17, 38)	32% (21, 42)	37% (26, 48)	32% (21, 42)	27% (17, 38)
Severe ankle injury (*n* = 31)	35% (19, 52)	45% (28, 63)	45% (28, 63)	42% (25, 59)	45% (28, 63)	45% (28, 63)	48% (31, 66)	55% (37, 72)

Prevalence is reported per 100 athletes.

All calculations are reported with 95% CIs.

#### Basketball performance markers in athletes with severe lower extremity injury

Performance metrics 1 and 2 y following severe lower extremity injury demonstrated decreased season minutes played [1 y: 0.5 (95% CI: 0.4, 0.6), *P* < 0.001; 2 y: 0.5 (95% CI: 0.3, 0.6), *P* < 0.001], season points scored [1 y: 0.5 (95% CI: 0.4, 0.7), *P* < 0.001; 2 y: 0.6 (95% CI: 0.4, 0.8), *P* < 0.001], and season rebounds [1 y: 0.6 (95% CI: 0.5, 0.7), *P* < 0.001; 2 y: 0.6 (95% CI: 0.5, 0.7), *P* < 0.001] compared to 1 y prior to severe injury.

When controlling for minutes played in a season, performance metrics 1 and 2 y following severe lower extremity injury demonstrated decreased season points scored per minute played [1 y: 0.5 (95% CI: 0.4, 0.6), *P* < 0.001; 2 y: 0.5 (95% CI: 0.4, 0.6), *P* < 0.001], and season rebounds per minute played [1 y: 0.5 (95% CI: 0.4, 0.6), *P* < 0.001; 2 y: 0.4 (95% CI: 0.3, 0.5), *P* < 0.001].

#### Sensitivity analyses

Including only severe knee injuries demonstrated similar results for minutes [1 y: 0.6 (95% CI: 0.4, 0.8); *P* < 0.001; 2 y: 0.5 (95% CI: 0.2, 0.7), *P* < 0.001], points [1 y: 0.6 (95% CI: 0.5, 0.8), *P* < 0.001; 2 y: 0.8 (95% CI: 0.6, 1.0), *P* < 0.001], and rebounds [1 y: 0.8 (95% CI: 0.6, 0.9), *P* < 0.001; 2 y: 0.6 (95% CI: 0.4, 0.7), *P* < 0.001] compared to the inclusion of all severe groin/hip/thigh, knee, and ankle injuries. Season points scored per minute played [1 y: 0.2 (95% CI: 0.1, 0.3), *P* < 0.001); 2 y: 0.6 (95% CI: 0.4, 0.8), *P* < 0.001], and season rebounds per minute played [1 y: 0.5 (95% CI: 0.4, 0.6), *P* < 0.001; 2 y: 0.4 (95% CI: 0.3, 0.6), *P* < 0.001] demonstrated similar results for 1 and 2 y following severe injury compared to the primary analyses. Using nonlinear analyses, basketball players 1 and 2 y following index year demonstrated decreased season minutes, points, and rebounds. Please refer to the Appendix S4 for nonlinear coefficients and figures.

## Discussion

This study found that only 196 (69%) of players participated in the NBA 1 y following a severe lower extremity injury, and only 66% of those players [135 (45% overall)] participated in the NBA for a second season following severe injury. Further, only 58 (30%) of NBA players returned to their preinjury number of games 1 y following injury and 48 (37%) returned to their preinjury number of games 2 y following injury. There were no differences in time to return to sport after ankle, knee, or thigh/hip/groin injuries, suggesting that injury time loss was similar, creating an analogous comparison of performance following injury. The first and second year after the injury NBA players observed nonlinear relationships to performance, and all groups demonstrated decreased performance following a severe lower extremity injury. The results of this study present a bleak picture of return to performance in the NBA.

Two thirds of basketball players who sustained a severe lower extremity injury participated at the NBA level 1 y following injury, with just under half participating 2 y following injury. These findings are similar compared to anterior cruciate ligament injuries in mens’ professional soccer (20). Further, NBA players who sustained an ACL tear, played on average 2 y less than healthy NBA controls (21). In another study evaluating Achilles tendon ruptures in NBA players, players who sustained an Achilles rupture were limited on average to two seasons following rupture, suggesting a potential survival effect (8). Player dropout was similar for groin/hip/thigh, knee, and ankle injuries. Initial dropout following severe injury may be due to the catastrophic nature of the injuries (20, 22). However, the further dropout of players in the second year likely indicates that returning to play alone is not sufficient, return to performance is necessary to continue a career in the NBA. It is possible that basketball players who remained in league play for 2 y may have greater basketball skill and performance than players who do not play in the NBA 2 y following a severe injury. However, further research is needed to investigate this hypothesis.

Most basketball players in this study did not return to their preinjury level of performance and demonstrated decreased performance 1 and 2 y following a severe lower extremity injury. These decreased performance findings were similar across groin/hip/thigh, knee, and ankle injuries. The results of this study support previous literature in basketball (6) and in sports such as baseball (23) and football (24). However, the previous literature instituted a case-control design, and compared to noninjured controls for only 1 y. In addition to the extended time to return to play after severe injury, it may require increased time to return to previous performance levels after returning to sport. Further, even if an athlete can perform at their preinjury level for a few games, after severe injury it is possible they are not able to sustain that level for an entire season (25). Another possible explanation is load management. Within the last decade, load management has almost universally been integrated into the NBA (26). Load management involves regulating minutes played with the aim of mitigating injury risk, decreasing the chance of missed competition (27), and ultimately increasing the chance of team success (5). Coaches, sports medicine, and performance professionals may rest or have stricter load management strategies for basketball players returning after severe injury. When evaluating performance metrics such as points per game or rebounds per game or per minute, NBA players also demonstrated decreased performance. Further research is needed to understand the association of different physical parameters, load management strategies, and return to preinjury performance in professional basketball.

### Practical applications

The custom R software package is open acces for convenient use. The authors hope that using a transparent reproducible methodology will help transform sports injury surveillance and prevention programs. These data are consistent with best practice for open-access data (28), based on ethical guidelines concerning the scientific process (29) and clinical reproducibility (30). Eleven years of basketball injury incidence, severity, and performance data are included, improving the stability and generalizability of these data. This study identified a potential prognostic factor in association to NBA performance. While this is only a preliminary investigation of one potential prognostic factor, aligned with PROGRESS recommendations (31), these raw open access data can be used by clinicians and coaches to better help create improved realistic prognostic recommendations for basketball player performance metrics following a severe lower extremity injury. For example, a basketball player can be matched through the player database and injury, and specific performance can be compared using the open access data and the subsequent code, providing tangible, real data applications and comparisons.

#### Limitations

As with all studies there are limitations. It should be noted only NBA data were extracted and analyzed, decreasing the ability to generalize these results other leagues or other countries. There is a risk of Type 1 and II error due to the multiple analyses performed and the moderate size of this sample. Due to these data being extracted from public sources, missing data cannot be truly quantified, decreasing the precision of these results. Nevertheless, randomized and blinded external data validation checks were executed with other public data to increase the interpretability of these results. Injuries may have been misclassified, and injuries were categorized to the nearest body part, decreasing the precision of these findings. Further, injuries were calculated from the first missed game. Injuries incurred in practice or training sessions could not be accurately determined, decreasing the precision of these results. Inclusion criteria consisted of NBA players who played at least three seasons in order to have substantial data to compare the effect of severe lower extremity injury on performance. Comparisons to healthy controls were not possible with these data, and decrease the interpretability of these results. Rookies were not included due to the paucity of performance data prior to index severe injury, and the potential effect of first time league play on performance and injury (13). However, NBA players may incur severe injuries that limit NBA play, causing a survival effect. Only seasonal performance data were available for analysis. Injuries may be sustained at different points throughout a season, causing different rehabilitation and return to play/performance timelines, decreasing the precision and interpretability of these results. Due to the volatility of game to game and in season player performance, these data should only be interpreted in context of the entire season performance. These data and analyses are designed to assess prognostic associations, causality cannot be determined from this study. Due to the data quality and methodology, causal structures and models could not be determined. As a result, the potential for collider bias inhibited covariates to be controlled for within the analyses, allowing only for unadjusted prognostic associations to be analyzed. However, the authors did perform further analyses, controlling for potential variables, including: age at primary severe injury, number of NBA seasons played the year of the primary severe injury, year of primary severe injury, body part (ankle, knee, or hip), and position (guard, forward, or center), and an interaction between age at primary severe injury, number of NBA seasons played during the year of primary severe injury. These results were similar to the primary analyses (Appendix S4).

### Conclusions

Fewer than half of basketball players who suffered a severe lower extremity injury were participating at the NBA level 2 y following injury. These results were similar for groin/hip/thigh, knee, and ankle injuries. Basketball players who sustained a severe groin/hip/thigh, knee, or ankle injury demonstrated decreased basketball performance across all performance metrics, for seasonal and per game metrics. Fewer than half of players were performing at previous preinjury levels 2 y following injury. Current sport injury data are inaccessible to the general clinician, athletes, or organizations outside of an individual team or league, inhibiting collaboration and transparent scientific enquiry. Suffering a severe lower extremity injury is potentially a prognostic factor in association to NBA performance. These data can assist sports medicine and performance professionals to educate and set performance expectations for players, teams, and organizations as they return to basketball following a severe lower extremity injury.

## Supplementary Material

pgac176_Supplemental_Files

## Data Availability

For the complete code and raw data obtained for this study, please refer to freely available online code and data repository: GitHub R Package “tylerferguson/NBAinjuries.”

## References

[1] Starkey C. 2000. Injuries and illnesses in the National Basketball Association: a 10-year perspective. J Athlet Train. 35:161. PMC1323413 16558626

[2] Drakos MC , Domb B, Starkey C, Callahan L, Allen AA. 2010. Injury in the National Basketball Association: a 17-year overview. Sports health. 2:284–290. 23015949 10.1177/1941738109357303 PMC3445097

[3] Bullock GS , et al. 2021. Temporal trends and severity in injury and illness incidence in the National Basketball Association over 11 seasons. Orthop J Sport Med. 9:23259671211004094. 10.1177/23259671211004094 PMC8207289 34179200

[4] Smith S. 2020. What is the real cost of injuries in professional sport?. Medium. [accessed 2020 July 6]. https://medium.com/@stephensmith_ie/what-is-the-real-cost-of-injuries-in-professional-sport-fee1d66a7502.

[5] Hägglund M , et al. 2013. Injuries affect team performance negatively in professional football: an 11-year follow-up of the UEFA Champions League injury study. Br J Sports Med. 47:738–742. 23645832 10.1136/bjsports-2013-092215

[6] Busfield BT , Kharrazi FD, Starkey C, Lombardo SJ, Seegmiller J. 2009. Performance outcomes of anterior cruciate ligament reconstruction in the National Basketball Association. Arthroscopy. 25:825–830. 19664500 10.1016/j.arthro.2009.02.021

[7] Harris JD , et al. 2013. Return-to-sport and performance after anterior cruciate ligament reconstruction in National Basketball Association players. Sports Health. 5:562–568. 24427434 10.1177/1941738113495788 PMC3806178

[8] Amin NH , et al. 2013. Performance outcomes after repair of complete Achilles tendon ruptures in National Basketball Association players. Am J Sports Med. 41:1864–1868. 23733634 10.1177/0363546513490659

[9] Ardern CL , et al. 2016. 2016 Consensus statement on return to sport from the First World Congress in Sports Physical Therapy, Bern. Br J Sports Med. 50:853–864. 27226389 10.1136/bjsports-2016-096278

[10] Drakos MC , Domb B, Starkey C, Callahan L, Allen AA. 2010. Injury in the National Basketball Association: a 17-year overview. Sports Health. 2:284–290. 23015949 10.1177/1941738109357303 PMC3445097

[11] Injury IOC , et al. 2020. International Olympic Committee consensus statement: methods for recording and reporting of epidemiological data on injury and illness in sports 2020 [including the STROBE extension for Sports Injury and Illness Surveillance (STROBE-SIIS)]. Orthop J Sport Med. 8:2325967120902908. 10.1177/2325967120902908 PMC7029549 32118084

[12] Landers RN , Brusso RC, Cavanaugh KJ, Collmus AB. 2016. A primer on theory-driven web scraping: automatic extraction of big data from the Internet for use in psychological research. Psychol Methods. 21:475. 27213980 10.1037/met0000081

[13] Martin CL , et al. 2021. Injury and illness incidence and influence of rookie season injury on career longevity in National Basketball Association players. JAMA Netw Open. 4: e2128199. 34605914 10.1001/jamanetworkopen.2021.28199 PMC8491104

[14] Ardern CL , et al. 2016. 2016 Consensus statement on return to sport from the First World Congress in Sports Physical Therapy, Bern. Br J Sports Med. 50:853–864. 27226389 10.1136/bjsports-2016-096278

[15] Borowski LA , Yard EE, Fields SK, Comstock RD. 2008. The epidemiology of US high school basketball injuries, 2005–2007. Am J Sports Med. 36:2328–2335. 18765675 10.1177/0363546508322893

[16] Bahr R , et al. 2020. International Olympic Committee Consensus statement: methods for recording and reporting of epidemiological data on injury and illness in sports 2020 [including the STROBE extension for Sports Injury and Illness Surveillance (STROBE-SIIS)]. Orthop J Sports Med. 8:232596712090290. 10.1177/2325967120902908 PMC7029549 32118084

[17] Orchard J , et al. 2010. Revision, uptake and coding issues related to the open access Orchard Sports Injury Classification System (OSICS) versions 8, 9 and 10.1. Open J Sports Med. 1:207. 10.2147/OAJSM.S7715 PMC3781871 24198559

[18] Hägglund M , Waldén M, Bahr R, Ekstrand J. 2005. Methods for epidemiological study of injuries to professional football players: developing the UEFA model. Br J Sports Med. 39:340–346. 15911603 10.1136/bjsm.2005.018267 PMC1725241

[19] Knowles SB , Marshall SW, Guskiewicz KM. 2006. Issues in estimating risks and rates in sports injury research. J Athlet Train. 41:207. PMC1472638 16791309

[20] Waldén M , Hägglund M, Magnusson H, Ekstrand J. 2016. ACL injuries in men’s professional football: a 15-year prospective study on time trends and return-to-play rates reveals only 65% of players still play at the top level 3 years after ACL rupture. Br J Sports Med. 50:744–750. 27034129 10.1136/bjsports-2015-095952

[21] Kester BS , Behery OA, Minhas SV, Hsu WK. 2017. Athletic performance and career longevity following anterior cruciate ligament reconstruction in the National Basketball Association. Knee Surg Sports Traumatol Arthrosc. 25:3031–3037. 26971105 10.1007/s00167-016-4060-y

[22] Casartelli NC , Leunig M, Maffiuletti NA, Bizzini M. 2015. Return to sport after hip surgery for femoroacetabular impingement: a systematic review. Br J Sports Med. 49:819–824. 25841163 10.1136/bjsports-2014-094414

[23] Peters SD , et al. 2018. The success of return to sport after ulnar collateral ligament injury in baseball: a systematic review and meta-analysis. J Shoulder Elbow Surg. 27:561–571. 29433647 10.1016/j.jse.2017.12.003

[24] Carey JL , Huffman GR, Parekh SG, Sennett BJ. 2006. Otcomes of anterior cruciate ligament injuries to running backs and wide receivers in the National Football League. Am J Sports Med. 34:1911–1917. 16870822 10.1177/0363546506290186

[25] Meredith SJ , et al. 2020. Return to sport after anterior cruciate ligament injury: Panther Symposium ACL Injury Return to Sport Consensus Group. Orthop J Sport Med. 8:2325967120930829. 10.1177/2325967120930829 PMC7328222 32647735

[26] Scamardella F , Russo N, Napolitano F. 2020. The phenomenon of load management. J Physical Educ Sport. 20: 2306–2309.

[27] Gabbett TJ , Jenkins DG. 2011. Relationship between training load and injury in professional rugby league players. J Sci Med Sport. 14:204–209. 21256078 10.1016/j.jsams.2010.12.002

[28] Bertagnolli MM , et al. 2017. Advantages of a truly open-access data-sharing model. N Engl J Med. 376:1178. 28328337 10.1056/NEJMsb1702054

[29] Alfonso F , et al. 2017. Data sharing: a new editorial initiative of the International Committee of Medical Journal Editors. Implications for the Editors’ Network. Cardiologia Croatica. 12:264–272. 10.4070/kcj.2016.0444 PMC5449522 28567078

[30] Baker M. 2016. 1,500 scientists lift the lid on reproducibility. Nature. 533:452–454. 27225100 10.1038/533452a

[31] Riley RD , et al. 2013. Prognosis Research Strategy (PROGRESS) 2: prognostic factor research. PLoS Med. 10:e1001380. 23393429 10.1371/journal.pmed.1001380 PMC3564757

