# Supplementary material for: Return to performance following severe ankle, knee, and hip injuries in National Basketball Association players
Source: PNAS Nexus. 2022 Sep 4;1(4):pgac176. doi: 10.1093/pnasnexus/pgac176 (PMC9802060; doi:10.1093/pnasnexus/pgac176)
Supplement: pgac176_Supplemental_Files [file pgac176_supplemental_files.zip › PNASNEXUS-PNASNEXUS-2022-00477-s01.docx]

## **Supplement**

## Appendix 1. Study Flow Chart


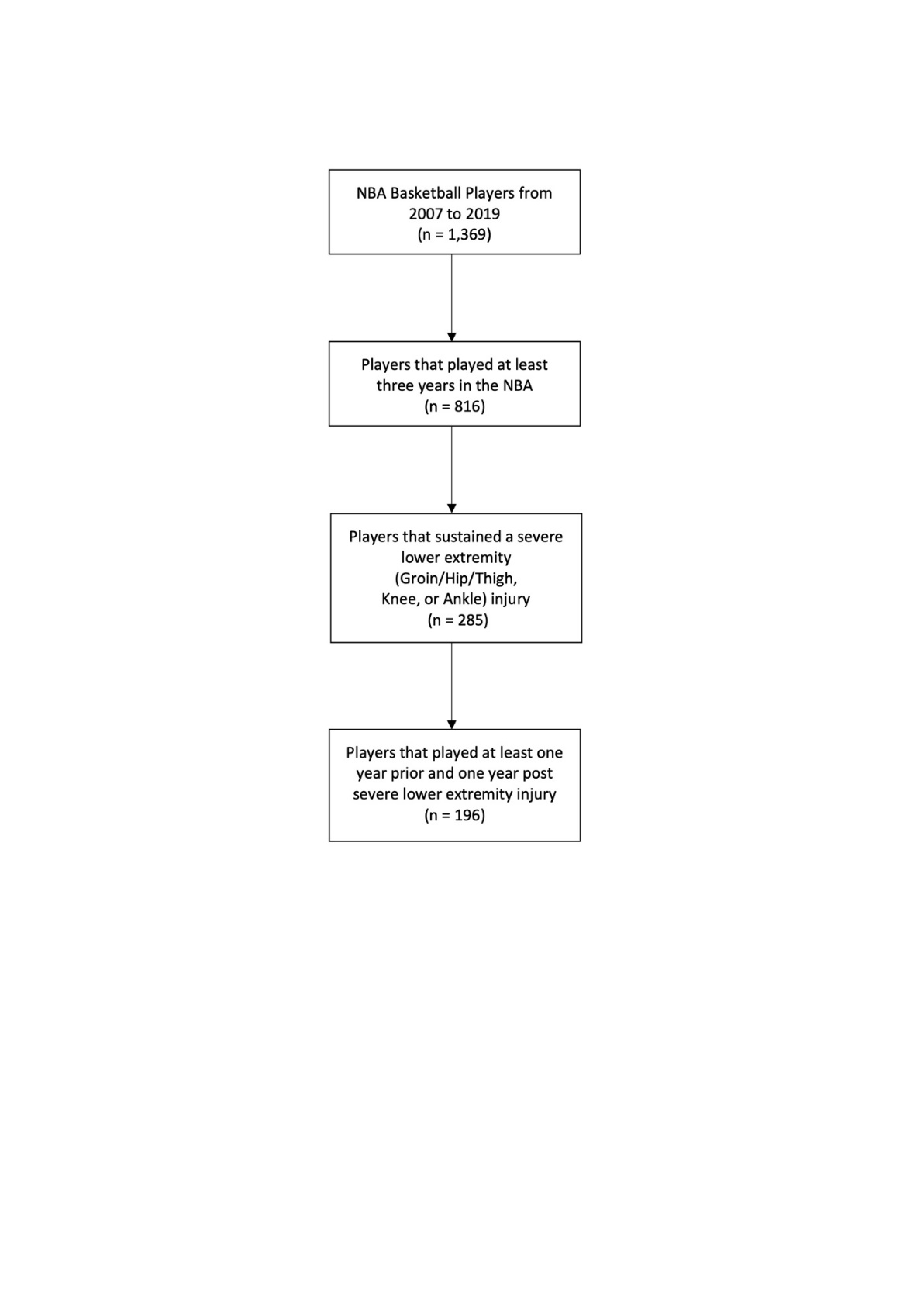


Appendix 2. Meaure of Agreement

| RandomNumberGenerated | idPlayerNBA | Season.x | groupPosition | Notes |
| --- | --- | --- | --- | --- |
| 799 | 201563 | 2010-11 | F |  |
| 1103 | 202344 | 2013-14 | F | Game missed sourced stated he did not dress 20th Jan - Data suggests injury 20th. Games was against detroit pistons 18th |
| 1166 | 202340 | 2013-14 | G | Did not play on 07/02/14, reported sat out - unable to find source date of injury |
| 1357 | 201978 | 2014-15 | F | Injury occured 13th April in Pelicans game - comfirmed 2 x sources |
| 1678 | 201596 | 2012-13 | G |  |
| 1996 | 202348 | 2012-13 | G |  |
| 2696 | 201936 | 2013-14 | F | Source said sprained 6/12/13, game review 13/12/13 stated aggravating a recent injury in the game and sitting out the 2nd half |
| 3709 | 203914 | 2016-17 | G |  |
| 3741 | 202699 | 2013-14 | F |  |
| 4093 | 1626178 | 2015-16 | F | Injury stated Saturday Practise 5th December 2015 |
| 4458 | 202704 | 2017-18 | G | Injury 26th December 2017 during game |
| 6349 | 202390 | 2013-14 | G | Unclear injury date - reports knee injury 24th developed into ankle. Bleacher report quotes injury as 22nd |
| 6474 | 203512 | 2016-17 | C | different number of games missed across sources DG - 5 in season games missed, injury 21/10/16 in pre season game |
| 6510 | 1717 | 2017-18 | C | Appears surgery date not specific injury date |
| 7180 | 2209 | 2007-08 | F | Incorrect date of injury DG - source reports injury happened 3rd Jan 2008 in practise, 10 games missed from injury date |
| 8538 | 101111 | 2007-08 | F | Incorrect injury date (19/12/2007) DG - Game recap reports injured practise 18th December 2007 |
| 8607 | 2548 | 2013-14 | G | 1 game missed according to this source DG -source reports 22/24th games missed. 22 report says sat out, 24th recap says ankle tweaked last week therefore missing game. Source reports injury occurred 19th March 2014 in celtics game |
| 2 | 201603 | 2010-11 | F | Only TSN source to confirm injury and date |
| 9132 | 1628391 | 2018-19 | F | Source doesn't report injury date in data, stated 8th that player was out with injury (not date of injury) stats show 4 x DNP from 3rd May |
| 94 | 200746 | 2008-09 | F | Injury 18/03 |
| 492 | 202970 | 2011-12 | F | "New Orleans is notoriously bad with injury reporting". Rotoworld mention he missed just one regular season game before being transferred (JV) DG - injury occured 24/4/12 |
| 2525 | 1627739 | 2017-18 | G | Injury reported in media on 18th Jan, but occurred on 17th during a game vs warriors. 18th was date of further evaluation and report |
| 3118 | 200752 | 2011-12 | F |  |
| 5536 | 101133 | 2017-18 | C |  |
| 5947 | 101139 | 2012-13 | F | Injury occurred one day before |
| 6355 | 202390 | 2014-15 | G | Concussion occured 2 days prior |
| 6601 | 203506 | 2015-16 | G | Injury 1 day prior (11/11) |
| 6848 | 101108 | 2010-11 | G | Injury 1 day prior |
| 8264 | 200748 | 2008-09 | F | Injury 1 day prior |
| 276 | 203937 | 2019-20 | F | Played on sore heel on 29th, 30th ruled out of next game |
| 419 | 1897 | 2007-08 | F | Unable to find another source for injury date, game sat out on 5th March, injury before this day |
| 986 | 101154 | 2009-10 | F | Correct date of surgery reported |
|  | 1628422 | 2017-18 | G | Unable to find official date of injury, date is when 1st game missed. 3 games misses not 4 |
| 2687 | 201936 | 2010-11 | G | Chronic issue, date is the game missed |
| 6636 | 979 | 2013-14 | F | 1st missed game 02/11, cannot say the injury did not happen on 1st, unable to find source |
| 7705 | 202338 | 2015-16 | C | Date is of missed game, game day decision |
| 8701 | 739 | 2012-13 | C | Stress injury, day to day assessment, eventually causing retirement |
| 9233 | 201156 | 2012-13 | G |  |
| 9318 | NA | NA | NA | Injury evening before 09/07, unable to find games missed |
| 176 | 2754 | 2013-14 | G |  |
| 1568 | 201960 | 2013-14 | F | Played game after injury - onset unclear |
| 2684 | 201936 | 2009-10 | G | Injury listed as day to day, reported on 21st - injury occurred 20/11 Dallas game |
| 3105 | 2200 | 2015-16 | C | Injury on 03/02 |
| 3145 | 1627771 | 2016-17 | G | 7 missed games according to tsn in this period, unable to find other date of injury, just of report |
| 3470 | 203110 | 2019-20 | F | Injury in game 1 day prior |
| 3987 | 201588 | 2010-11 | G | 1 game missed |
| 7119 | 101179 | 2009-10 | G | Injury 10th March |
| 8642 | 203079 | 2013-14 | G | Chronic injury, no start date, only date of 1st missed game DG injury happened 22/11 |
| 8969 | 101114 | 2010-11 | G | injury on 26/01 |
| 705 | 201587 | 2011-12 | F | injury 07/04 |
| 1249 | 201166 | 2015-16 | G | injury 18/11 |
| 2989 | 201568 | 2011-12 | F | Incorrect injury classification - ankle |
| 3225 | 1938 | 2012-13 | G | Ongoing problem when reported DG missed games not correct from injury |
| 3262 | 203497 | 2016-17 | C | 13th is date of 2nd missed game, unsure of date of injury |
| 3472 | 203475 | 2014-15 | G | Played 1 minute 9th feb then removed due to injury |
| 3686 | 2734 | 2010-11 | G | Injury day before |
| 3826 | 202330 | 2013-14 | F | injury in a game on 07/01 |
| 3869 | 201945 | 2016-17 | G | Ongoing labral injury, quoted date is the missed game |
| 4491 | 1744 | 2008-09 | C | no data found, described a strained quadricep in pre-season |
| 5172 | 201951 | 2016-17 | G | Injury 1 day prior |
| 5719 | 201564 | 2014-15 | G | problems with hamstring prior to this date |
| 6316 | 959 | 2008-09 | G | Injured 26/11 |
| 6389 | 2749 | 2015-16 | G |  |
| 6495 | 1717 | 2012-13 | F | injury 29th |
| 6662 | 203124 | 2017-18 | C |  |
| 6779 | 2225 | 2016-17 | G | Injury in game 03/05 |
| 8022 | 978 | 2007-08 | F | date of injury unclear - appears injured, played, re injured. 3rd game missed 20/12 |
| 9325 | NA | NA | NA | date of surgery 31/07 |
| 1116 | 200767 | 2007-08 | F |  |
| 1369 | 203493 | 2018-19 | G | neck injury reported 15/01/20, foot injury 4/4/19 |
| 2274 | 2736 | 2015-16 | F | Injury on 20th Jan. Unabkle to open website due to foreign IP adress, however sample article mentioned injury in wednesdays game (published 22nd Jan) |
| 2549 | 1628979 | 2018-19 | G |  |
| 2578 | 201961 | 2015-16 | G |  |
| 2791 | 202324 | 2017-18 | C |  |
| 3487 | 203210 | 2016-17 | F |  |
| 3494 | 203210 | 2018-19 | F | Injury 19/10, 20th was surgery, 12 games missed |
| 4270 | 2743 | 2016-17 | F | spasms reported 08/04 |
| 5507 | 1894 | 2008-09 | F | Injury on 24th March, missed 3 games (played a game then missed 7 more games) |
| 181 | 2754 | 2014-15 | G |  |
| 516 | 202337 | 2016-17 | F |  |
| 1713 | 2199 | 2018-19 | C |  |
| 2008 | 203109 | 2012-13 | F |  |
| 2521 | 2399 | 2015-16 | F |  |
| 2773 | 202324 | 2010-11 | F |  |
| 2843 | 201178 | 2009-10 | C | 6 games missed from 4/12/09, other source reports 3 games missed due to illness. Virus reported 08/12 |
| 3392 | 1626203 | 2016-17 | G | No classification |
| 3914 | 1626209 | 2018-19 | F | Only 1 missed game from illness date |
| 4703 | 2207 | 2014-15 | G | Reported unable to breathe because of illness 04/12 |
| 4826 | 203093 | 2015-16 | F | illness reported 28/12 |
| 5046 | 2594 | 2016-17 | G | Confirmed date but not illness |
| 5955 | 101139 | 2014-15 | G | confirmed date but not illness |
| 5960 | 101139 | 2015-16 | F |  |
| 6244 | 2211 | 2008-09 | F | virus sourced as 17/12/08 |
| 6799 | 202718 | 2014-15 | F |  |
| 7207 | 201576 | 2010-11 | C | 11 did not play since 9/1/11 - only 11/1/11 where the player did not dress. Other source reports 3 games missed due to illness |
| 8337 | 201977 | 2013-14 | G |  |
| 8817 | 101110 | 2013-14 | F |  |
| 9086 | 200766 | 2008-09 | G | not 11 games missed post illness |
| 189 | 2754 | 2017-18 | F |  |
| 566 | 2571 | 2014-15 | G | injured in game on 25th |
| 1455 | 101115 | 2010-11 | C | player missed game to suspension x 3. 1 game due to knee, 4 due to suspension |
| 2049 | 203552 | 2018-19 | G | injured 14/11/18 |
| 2247 | 203521 | 2017-18 | G |  |
| 2785 | 202324 | 2016-17 | F |  |
| 3038 | 708 | 2008-09 | F |  |
| 3346 | 201569 | 2012-13 | G |  |
| 3361 | 201569 | 2017-18 | G |  |
| 3365 | 201569 | 2019-20 | G |  |
| 4306 | 2738 | 2011-12 | F |  |
| 4721 | 1626169 | 2018-19 | F |  |
| 5120 | 1629641 | 2019-20 | G | Injury on 13/10 in preseason game, missed opening game then reassigned to G league DG - 3 games missed, played 30/10/19 and then Dec 2019 |
| 6987 | 2554 | 2008-09 | G |  |
| 7075 | 1899 | 2008-09 | G | Injury 25th April game 4 1st round play offs, continued playing. Surgery reported 26th may |
| 7144 | 2419 | 2013-14 | F | injury 17/12 |
| 8006 | 2052 | 2011-12 | F | 8 games missed, data quotes returns after 7 days which is incorrect. Injury occurred much earlier and was a problem throughout January as well as february - DG 8 games missed from 31/01/12 sourced as sore knee date |
| 8203 | 2041 | 2008-09 | C | Injury on 12/01 day prior |
| 8512 | 2760 | 2013-14 | C | Injury 26/01 DG- 3 games from 31/01/14 |
| 8907 | 202355 | 2017-18 | C | Injury in game wednesday 18/02 |
| 87 | 202332 | 2014-15 | C | INjured 28/01/15 in Oklahoma game |
| 1191 | 1882 | 2012-13 | C |  |
| 1220 | 200758 | 2013-14 | F |  |
| 1984 | 2037 | 2014-15 | G | Injury 2/3/15 in Minnesota game |
| 2089 | 956 | 2011-12 | C | One source saysr 4missed, on BBall Ref 6 games missed since injury |
| 2267 | 2736 | 2013-14 | F |  |
| 3982 | 2403 | 2018-19 | C | 21 games missed. Calf Injury reported since start of october in preseason |
| 6891 | 1626166 | 2018-19 | G | Injured 10/12/18. |
| 8112 | 1628369 | 2018-19 | F | missed game as not with the team. injury on 07/04 |
| 8970 | 101114 | 2011-12 | G | Aggravated injury on 09/03 |
| 696 | 1499 | 2008-09 | F |  |
| 123 | 203145 | 2017-18 | G |  |
| 903 | 204021 | 2014-15 | C |  |
| 1294 | 2198 | 2010-11 | C |  |
| 2339 | 203915 | 2016-17 | G |  |
| 2475 | 2768 | 2009-10 | G |  |
| 3153 | 202087 | 2016-17 | F |  |
| 3600 | 201946 | 2015-16 | F |  |
| 3626 | 201935 | 2015-16 | G |  |
| 4064 | 1628988 | 2018-19 | G |  |
| 4233 | 1607 | 2007-08 | G |  |
| 4247 | 1716 | 2011-12 | G |  |
| 5022 | 1627788 | 2018-19 | G |  |
| 5525 | 101133 | 2010-11 | C |  |
| 6175 | 201043 | 2007-08 | C |  |
| 6199 | 203102 | 2012-13 | F |  |
| 6209 | 202389 | 2013-14 | C |  |
| 6720 | 2457 | 2013-14 | G |  |
| 8564 | 2063 | 2008-09 | C |  |
| 9135 | 203966 | 2017-18 | F |  |
| 2634 | 203516 | 2015-16 | F | Not 5 games missed in a row - no report online other than illness |
| 2996 | 201568 | 2014-15 | F |  |
| 3585 | 1888 | 2011-12 | G | Injured 5/3/12 - injury confirmed |
| 5214 | 101135 | 2013-14 | F |  |
| 5642 | 2755 | 2011-12 | G | only 24 games missed (to the end of the season - played first game of the next season) - Injured 11/03/12 |
| 6345 | 202390 | 2011-12 | G | reports only 2 games missed x 2 sources. Injury 23/03 |
| 6629 | 979 | 2011-12 | C |  |
| 7910 | 2747 | 2018-19 | G | Injured 16/10/18 |
| 9162 | 101120 | 2007-08 | G |  |
| 9273 | 203469 | 2016-17 | C |  |
| 81 | 203128 | 2014-15 | F | COnfirmed missed game and injury but did not specify time of injury event checking previous match recap. injury 14/03, 1 game missed |
| 755 | 203382 | 2015-16 | C |  |
| 1524 | 948 | 2011-12 | C | confirmed injured in game 15/4/12 |
| 2681 | 2501 | 2013-14 | F |  |
| 4505 | 2544 | 2014-15 | F |  |
| 5009 | 202688 | 2015-16 | G |  |
| 5078 | 1628398 | 2018-19 | F | Injury 2/1/19 |
| 5346 | 2733 | 2012-13 | G | Unable to find another source for date of injury |
| 6081 | 1737 | 2009-10 | C |  |
| 6329 | 959 | 2013-14 | G | taken out of game 10th November due to injury |
| 6388 | 2749 | 2015-16 | G |  |
| 6497 | 1717 | 2014-15 | F | Reported as precautionary rest from game - injury not identified |
| 6788 | 2225 | 2018-19 | G | Injury 23/11/18. Injury assumed 21/11 |
| 7278 | 200755 | 2017-18 | G |  |
| 7481 | 201565 | 2016-17 | G | Injury date not confirmed (chronic back issue from news missing game recently). Injury 13/12 |
| 7607 | 202336 | 2010-11 | C |  |
| 8104 | 2741 | 2008-09 | C | Swift known to have back issues in his career, cannot match dates |
| 8573 | 202696 | 2014-15 | C |  |
| 8816 | 101110 | 2013-14 | F |  |
| 9101 | 1629057 | 2018-19 | C | Player missed 7 games - missed 3 played 1 missed 4 more |

Appendix 3. National Basketball Association Players Performance Statistics for One Year Prior and One and Two Years Following Severe Injury,Stratified by Groin/Hip/Thigh, Knee, and Ankle Injuries

| Variable | Severe Groin/Hip/Thigh Injury  (n = 39) | | | Severe Knee Injury  (n = 111) | | | Severe Ankle  Injury  (n = 46) | | |
| --- | --- | --- | --- | --- | --- | --- | --- | --- | --- |
|  | One Year  Prior | One Year Following | Two Years Following | One Year  Prior | One Year Following | Two Years Following | One Year  Prior | One Year  Following | Two years Following |
| Games Played | 64 (24) | 55 (24) | 56 (26) | 64 (25) | 50 (25) | 55 (25) | 66 (24) | 48 (24) | 56 (21) |
| Games Started | 41 (29) | 28 (29) | 36 (30) | 40 (30) | 27 (31) | 33 (32) | 37 (27) | 22 (27) | 31 (29) |
| Season Minutes Played | 1746 (881) | 1412 (881) | 1511 (917) | 1746 (893) | 1231 (893) | 1396 (971) | 1677 (857) | 1131 (858) | 1412 (822) |
| Minutes Played Per Game | 25.7 (8.8) | 22.7 (9.4) | 25.1 (9.0) | 26.2 (8.1) | 21.5 (9.0) | 23.2 (10.2) | 24.8 (7.4) | 20.7 (9.5) | 23.2 (8.9) |
| Season Points | 782 (501) | 628 (501) | 708 (499) | 779 (522) | 562 (522) | 690 (614) | 704 (477) | 512 (477) | 678 (554) |
| Points Per Game | 11.2 (6.0) | 9.3 (6.2) | 11.7 (6.1) | 11.6 (6.2) | 9.3 (6.4) | 10.6 (7.4) | 10.4 (4.6) | 8.9 (5.8) | 10.6 (6.9) |
| Season Rebounds | 298 (214) | 224 (217) | 268 (214) | 313 (206) | 237 (212) | 249 (212) | 259 (170) | 208 (189) | 260 (209) |
| Rebounds per Game | 3.6 (2.6) | 2.7 (2.6) | 4.6 (3.6) | 3.8 (2.5) | 2.9 (2.6) | 3.9 (2.7) | 3.2 (2.1) | 2.5 (2.3) | 4.2 (2.6) |

Results are reported as mean (standard deviation)

Appendix 4. Non-Linear Analyses

*Season Minutes*

There was a non-linear relationship between the season minutes in the season prior to sustaining a severe lower extremity injury and the season following a severe lower extremity injury:

- Knot 1 (≤1460 season minutes) showed in unadjusted analysis a positive relationship (0.2 (95% CI: 0.1, 0.4), p = 0.019); Adjusted: 0.3 (95% CI: 0.1, 0.5), p = 0.006);
- Knot 2 (1461 to 1750 season minutes): Unadjusted 0.3 (95% CI: -0.2, 0.7), p = 0.240; Adjusted: 0.2 (95% CI: -0.2, 0.5), p = 0.457);
- Knot 3: (> 1750 season minutes) Unadjusted 1.0 (95% CI: -1.4, 3.5), p = 0.406; Adjusted: 0.7 (95% CI: -1.6, 2.9), p = 0.570).

There was a non-linear relationship between the season minutes in the season prior to sustaining a severe lower extremity injury and two seasons following a severe lower extremity injury:

- Knot 1 (≤1460 season minutes): Unadjusted: -0.4 (95% CI: -0.6, -0.1), p < 0.001); Adjusted: -3.3 (95% CI: -5.5, -0.1), p = 0.004);
- Knot 2 (1461 to 1750 season minutes): Unadjusted: 1.0 (95% CI: 0.5, 1.6), p < 0.001; Adjusted: 1.0 (95% CI: 0.6, 1.4), p < 0.001);
- Knot 3: (> 1750 season minutes) Unadjusted: -1.6 (95% CI: --4.5, 1.1), p = 0.234; Adjusted -2.4 (95% CI: -4.9, 0.1), p = 0.059).

### **
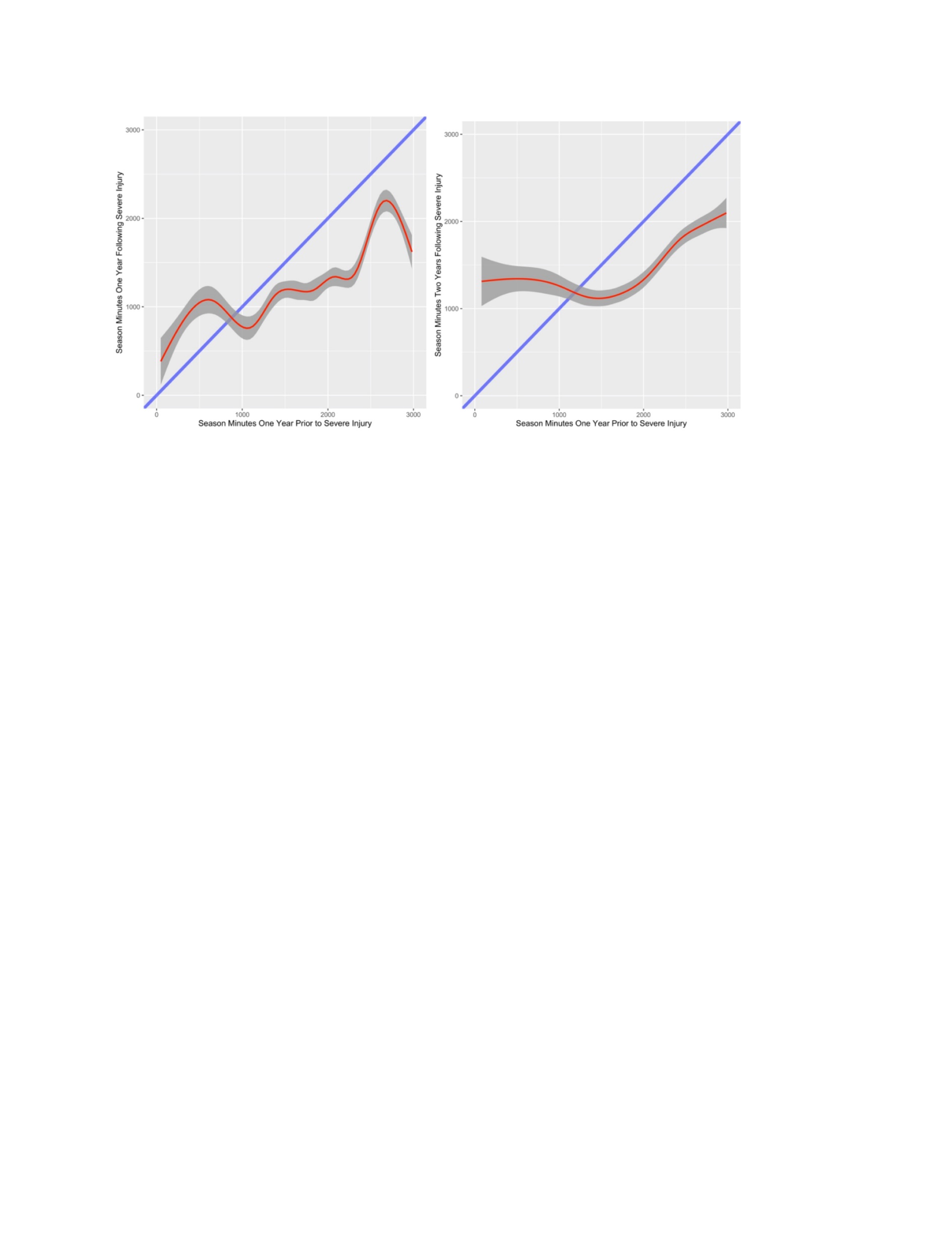
**

Figure 1. Comparison of Season Minutes of the Season Prior and Seasons One and Two Following Lower Extremity Injury.>

*The 45-degree line demonstrates perfect performance between season prior to a severe injury and season following severe injury

### *Season Points*

A nonlinear relationship was observed between season points in the season previous to severe lower extremity injury and season following severe lower extremity injury (Figure 1 A and B):

- Knot 1 (≤534 season points): Unadjusted 0.3 (95% CI: 0.1, 0.5), p = 0.011; Adjusted: 1.0 (95% CI: 0.1, 1.8), p = 0.027;
- Knot 2 (535 to 769 season points): Unadjusted: 1.0 (95% CI: 0.1, 1.9), p = 0.035; Adjusted: 0.7 (95% CI: -0.2, 1.6), p = 0.129;
- Knot 3 (>769 season points): Unadjusted: -2.0 (95% CI: -4.8, 0.8), p = 0.164; Adjusted: -1.0 (95% CI: -3.6, 1.6), p = 0.439).

A nonlinear relationship was observed between season points in the season previous to severe lower extremity injury and two seasons following severe lower extremity injury:

- Knot 1 (≤534 season points): Unadjusted: -0.6 (95% CI: -1.0, -0.3), p < 0.001; Adjusted: -0.6 (95% CI: -0.8, -0.3), p < 0.001;
- Knot 2 (535 to 769 season points): Unadjusted: 4.1 (95% CI: 2.8, 5.3), p < 0.001; Adjusted: 3.4 (95% CI: 2.3, 4.5), p < 0.001;
- Knot 3 (>769 season points): Unadjusted: -9.8 (95% CI: -13.4, -6.2), p < 0.001; Adjusted -8.0 (95% CI: -1.1, -4.8), p < 0.001).


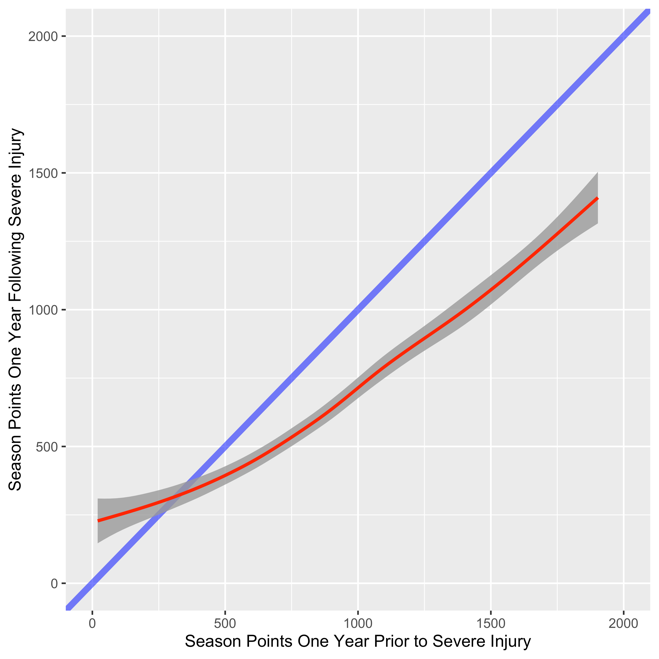

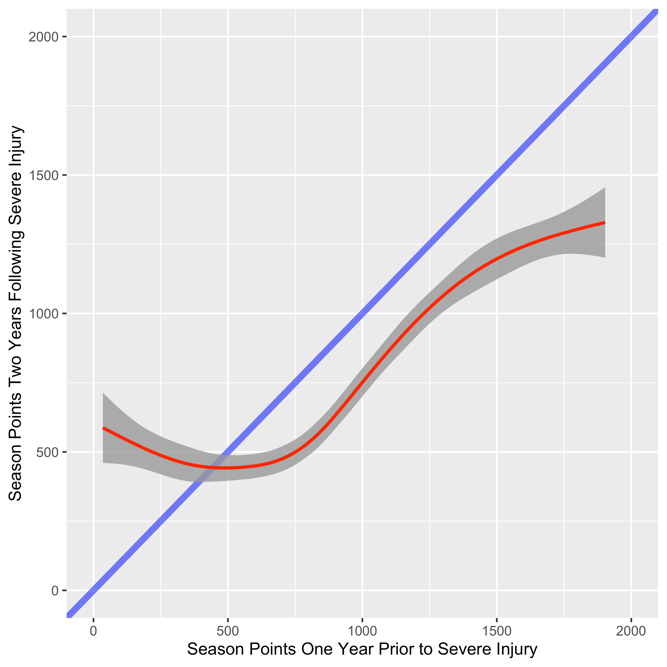


**Figure 2**. Comparison of Season Points the Season Prior and One and Two Seasons Following a Severe Lower Extremity Injury.

*The 45-degree line demonstrates perfect performance between season prior to a severe injury and season

*Season Rebounds*

There was no non-linear relationship between rebounds the season prior to severe lower extremity injury and the season following a severe lower extremity injury, as only knot 2 was significant; however, non-linear analyses demonstrated the best aikike’s information criterion and least error:

-Knot 1: (< 115 Rebounds): Unadjusted: 0.06 (95% CI: -0.1, 0.3), p = 0.562; Adjusted: 0.1 (95% CI: -0.1, 0.3), p = 0.361;

-Knot 2: (115 to 370 Rebounds): Unadjusted: 2.0 (95% CI: 0.9, 3.1), p < 0.001; Adjusted: 1.5 (95% CI: 0.5, 2.6), p = 0.005;

-Knot 3: (>370 Rebounds): Unadjusted: -3.9 (95% CI: -6.8, -0.9), p = 0.010; Adjusted: -2.5 (95% CI: -5.4, 0.4), p = 0.093

There was no non-linear relationship between rebounds the season prior to severe lower extremity injury and two season following a severe lower extremity injury, as only knot 1 was significant; however, non-linear analyses demonstrated the best aikike’s information criterion and least error:

-Knot 1: (< 115 Rebounds): Unadjusted: 0.4 (95% CI: 0.2, 0.6), p < 0.001; Adjusted: 0.4 (95% CI: -0.4, 1.1), p = 0.389;

-Knot 2: (115 to 370 Rebounds): Unadjusted: 0.4 (95% CI: -0.4, 1.3), p = 0.289; Adjusted: 0.4 (95% CI: -0.4, 1.1), p = 0.389;

-Knot 3: (>370 Rebounds): Unadjusted: -0.5 (95% CI: -3.1, 2.2), p = 0.164; Adjusted: 0.3 (95% CI: -2.7, 2.2), p = 0.844

##
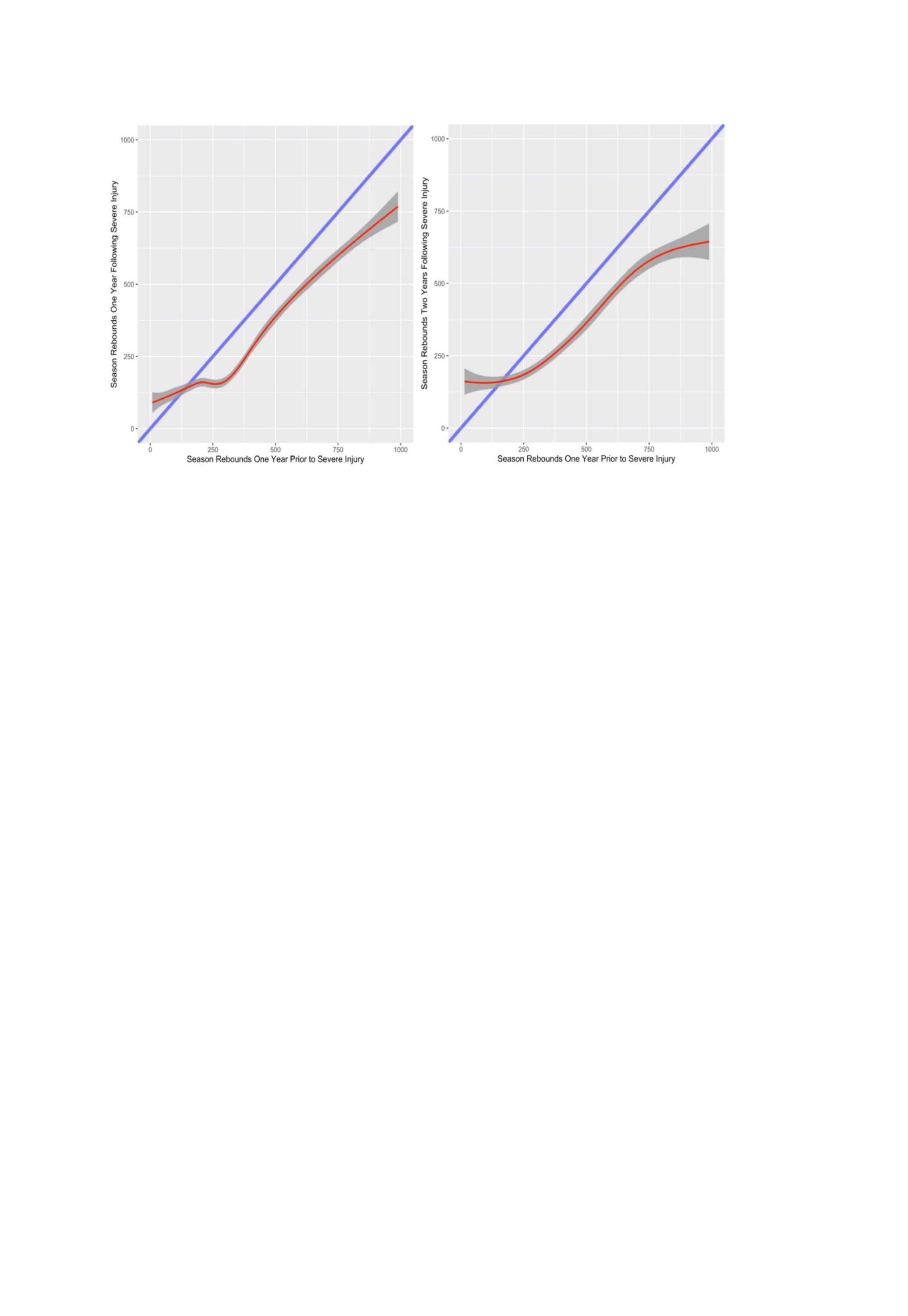


**Figure 3**. Comparison of Season Rebounds Attempted the Season Prior and One and Two Seasons Following a Severe Lower Extremity Injury.

*The 45-degree line demonstrates perfect performance between season prior to a severe injury and season following severe injury.

Appendix 4. Controlling for covariates, primary analyses

Performance metrics one and two years following severe lower extremity injury demonstrated decreased season minutes played (One Year: 0.4 (95% CI: 0.3, 0.5), p <0.001); Two Years: 0.3 (95% CI: 0.2, 0.5), p < 0.001), season points scored (One Year: 0.5 (95% CI: 0.4, 0.7), p < 0.001; Two Years: 0.6 (95% CI: 0.4, 0.7), p < 0.001), and season rebounds (One Year: 0.5 (95% CI: 0.4, 0.6), p < 0.001; Two Years: 0.5 (95% CI: 0.4, 0.6), p < 0.001) compared to one year prior to severe injury.

When controlling for minutes played in a season, performance metrics one and two years following severe lower extremity injury demonstrated decreased season points scored per minute player (One Year: 0.3 (95% CI: 0.2, 0.4), p <0.001); Two Years: 0.3 (95% CI: 0.2, 0.4), p < 0.001), and season rebounds per minute played (One Year: 0.4 (95% CI: 0.3, 0.5), p <0.001); Two Years: 0.3 (95% CI: 0.2, 0.4), p < 0.001)
